# Biomining of metals: new challenges for the next 15 years

**DOI:** 10.1111/1751-7915.13985

**Published:** 2021-11-30

**Authors:** Patricio Martínez‐Bellange, Diego von Bernath, Claudio A. Navarro, Carlos A. Jerez

**Affiliations:** ^1^ Biomining Consultant Santiago Chile; ^2^ Laboratory of Molecular Microbiology and Biotechnology Department of Biology Faculty of Sciences University of Chile Santiago Chile

## Abstract

Due to the current and future scenario in which phenomena such as global warming, massive industrial waste, excessive pollution of the ecosystem, water scarcity, among other negative variables, our planet and society, faces the urgent need to advance in the generation of more sustainable and environmentally friendly mining methods. The decline in the quality of the geological resources, specifically the increase of low‐grade minerals, has created a scenario under which mining companies must make great efforts to maintain their current production levels.

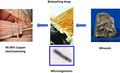

Due to the current and future scenario in which phenomena such as global warming, massive industrial waste, excessive pollution of the ecosystem and water scarcity, among other negative variables, our planet and society face the urgent need to advance in the generation of more sustainable and environmentally friendly mining methods. Because of this, the international community is demanding the use of latest generation tools and new ways of mining. On the other side, the new low‐carbon and renewable energy resources are demanding metals and minerals in an exponential way. But the extraction of these critical elements, such as copper, cobalt, nickel and rare earth elements REE, among others, is becoming every year more difficult and energy demanding. The decline in the quality of the geological resources, specifically the increase in low‐grade minerals, has created a scenario under which mining companies must make great efforts to maintain their current production levels.

Also, the deterioration of ore quality requires more complex processing schemes and strategies to manage the presence of complex mineralogy and complex contaminants. All these factors are stimulating the need for a strong development of innovative and sustainable technologies to ensure the natural elements’ production is compatible with environmental regulations, which will command the next years of the mining industry, pushing it to circular economy strategies.

Parallel to this, the exponential advances in biotechnology and applied microbiology within the last 40 years have captured the attention of the mining industry, in order to explore how to solve some of the above‐mentioned challenges and defining the concept of biomining (Johnson, 2014). One of these technologies that has been under development in different mining countries is bioleaching (Jerez, 2017a,b) (Fig. 1).

**Fig. 1:**
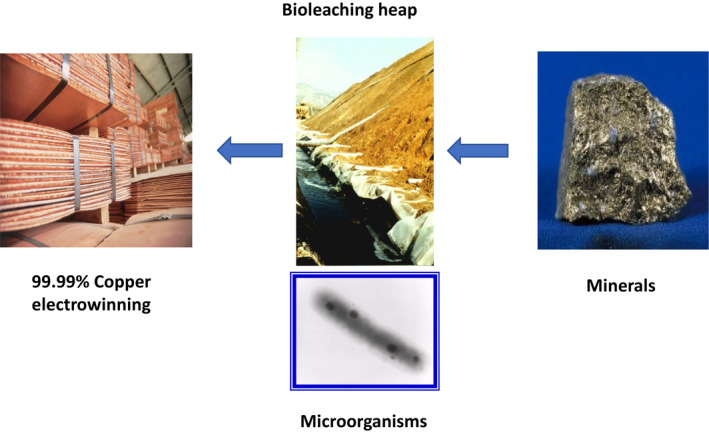
A simplified scheme illustrating how to obtain copper by using bioleaching of chalcopyrite.

The most known application for this technology is the bio‐oxidation of copper sulphides from primary resources, but opportunities in the mining industry will target too the processing of secondary resources, such as tailings, slag, concentrates and dumps as well as run of mine and crushed low‐grade ore material, with an increasingly positioning of bioleaching as a natural solution perfectly compatible with social demands of sustainability (Kaksonen *et al*., 2020).

Also, in the mining industry today, it is well‐accepted that bioleaching has a lower cost over alternatives in the treatment of low‐grade ore but is important to make an emphasis in the right operational conditions to have good performance of the process with the understanding that ‘microorganisms are not as rocks’. This economical advantage mentioned makes possible the economic extraction of marginal metal grade ore and, therefore, increases the mining reserves with prospecting longer mining plans under sustainable conditions.

As was mentioned before, additional application of bioleaching may focus on the re‐processing of fresh or abandoned tailings, as it allows valorisation of current environmental liabilities, eliminating a great headache for many companies and authorities given the great impact they generate on the ecosystem. This approach links with circular economy and zero mining waste policies, giving the mining industry an important seal of sustainability, extracting critical elements for world industry, such as REE, copper, cobalt, nickel and magnesium, among others.

Bioleaching is also under current interest for treatment of complex concentrates in copper production that contain appreciable amounts of toxic elements, such as arsenic, which currently either cannot be processed by smelters because of emissions regulation or are sold to markets with significant economical penalties. The use of bioleaching for this purpose could make possible the economical production of copper‐enriched liquors, leaving behind the contaminants to be neutralized with different applications.

An interesting approach to have a non‐disturbing mining process with zero waste production has also pushed the evaluation of *in situ* bioleaching. This technology, adapted from *in situ* leaching (ISL), proposes the drilling in the ore vain with injection of solutions with microorganisms to leach the value elements from the ore, without blasting, crushing or grinding. In this aspect, still being under discussion how this technology can be controlled in some biochemical aspects (Vargas *et al*., 2020) and in a very critical aspect, avoiding the potential percolation and contamination of groundwater. The last will be a big challenge for a real sustainable application of this technology.

Other applications of biomining, with less development at date, have focus into switching the current mining technologies that demand chemicals for selective ore separation, to a biomimicry approach, using biological strategies for these purposes. There are different alternatives, using cells (biosorption), peptides, viruses and metabolites as ‘selective binders’ for directed extraction of metals and in other cases for removal of contaminants from mining solutions or ore concentrates (Pollmann *et al*., 2016; Levett *et al*., 2021).

All these developments seem to be real sustainable technologies for mining, which can turn the old and contaminating ones, into clean and environmental respectful new technologies to be applied in the next 15 years. The challenge is running, and the opportunity for the mining industry is at hand.

To tackle these hydrometallurgical challenges in a sustainable manner, water must be used efficiently. Water is essential for mining operations; it is needed in the mine as well as in the ore processing and valuable metals recovery. Generally, continental water is preferred and used, but recently mining companies have been sourcing some of their water from the ocean. Due to stricter environmental policies, social pressure and technology advances, it is now technically and economically possible to use sea water, untreated or with some degree of desalination.

The implementation of sea water by the mining industry poses some challenges, such as the feasibility of transportation from the coast to mine sites in the mountains, the presence of ions that can hinder metallurgical processes or the effect of chloride on the structures and the different processes involved. Therefore, the use of sea water either plain or desalinated is subject to a technical–economical evaluation, which has been proven to be cost effective in several projects (Lagos *et al*., 2018).

Noteworthy is the case of BHP’s Escondida mine, the biggest copper mine in the world, which has vowed to stop using continental waters by 2030, becoming fully dependent on sea water obtained from their coastal desalination plants. This goes in line with the evermore stricter environmental policies enforced by authorities and local communities. It is expected that the whole industry shifts towards the same goal, as it has been shown during recent years where the consumption of continental water has either dropped or being sustained, and both the recirculation of waters and consumption of seawater have increased (Montes, 2019).

Most of the seawater used by the industry is and will be desalinated (Montes, 2019), which is a great advancement as it put less pressure on continental water, but it also adds a new layer of potential environmental and social conflict in the coastal sites. To fulfil their water requirements, industry has installed large desalination plants, which use filtering technologies, such as reverse osmosis, to produce fresh water. One of the downsides of this plants is that they generate large amounts of brines which must be properly disposed to avoid any environmental problems. Also, desalination is an energy‐intensive process; so to avoid any conflict or possible future regulations, mining companies need to consider the origin of the energy utilized. Thus, it is of great interest to develop alternatives that are less water intensive to process copper sulphides, hopefully with partial or no desalination.

Bioleaching appears as an interesting alternative for the following reasons: (i) being a hydrometallurgical process, it consumes less water than concentration, (ii) the depletion of oxides will leave some hydrometallurgical plants with spare capacity that might be converted to bioleaching operations and (iii) it can be cheaper to operate than other alternatives. Considering that using seawater might become the new standard and that sulphides are more prone to be leached in acid chloride medium (Davis‐Belmar *et al*., 2014; Bobadilla‐Fazzini *et al*., 2017), it might be interesting to study how bioleaching can be used in such scenario. It is a well‐documented fact that acidophiles are very sensitive to the presence of chloride and that such effect might be enhanced when combined with copper (Falagán and Johnson, 2018), so further research is needed to study the technical and economic factors for applying bioleaching in high acid chloride conditions.

Finally, in the near or not‐so‐near future, space biomining is of great interest. Colonization of other near planets will require enhancing biomining, and bioleaching for asteroid and planetary deployment has been indicated as one of many important synthetic biology possibilities (Menezes *et al*., 2015).

Microorganisms are used to economically biomine not only copper and other metals but also (REEs) required for electronic industries and alloy production. Cockell *et al*., 2020, have demonstrated that these REEs can be extracted in simulated microgravity and Mars gravity. No significant difference in final yields was observed between gravity conditions, showing the efficacy of the process under different gravity regimens. These data demonstrate the potential for space biomining and the principles of a reactor to advance human industry and mining beyond Earth. The authors concluded their results demonstrate the biological mining of economically important elements in space and in different extraterrestrial gravity environments. They have also demonstrated the principles of a miniature space biomining reactor and as they point out microbe–mineral interactions for advancing the establishment of a self‐sustaining permanent human presence beyond the Earth and the technical means to do that (Cockell *et al*., 2020). It is expected these, and other similar advances will not only avoid the great contaminations that have been generated by humans to obtain metals in planet Earth but rather generate a totally sustainable microbial ‘planetary mining’ (Cockell *et al*., 2020).

## Conflict of interest

None declared.
